# Editorial: Emerging treatments and approaches for moral injury and moral distress

**DOI:** 10.3389/fpsyt.2023.1125161

**Published:** 2023-01-17

**Authors:** Eric Vermetten, Chelsea Jones, Lorraine Smith MacDonald, Jackie June ter Heide, Andrew James Greenshaw, Suzette Brémault-Phillips

**Affiliations:** ^1^Department of Psychiatry, Leiden University, Leiden, Netherlands; ^2^Heroes in Mind Advocacy and Research Consortium, Faculty of Rehabilitation Medicine, University of Alberta Edmonton, Edmonton, AB, Canada; ^3^ARQ National Psychotrauma Centre, Diemen, Netherlands; ^4^Department of Psychiatry, Faculty of Medicine and Dentistry, University of Alberta, Edmonton, AB, Canada

**Keywords:** moral injury, PTSD, trauma, treatment, moral distress

## Advanced exploration of interventions for moral injury and moral distress

Current evidence-based therapies to treat trauma-affected populations, especially military members and first responders, have had variable success. Treatment response may be impeded by a lack of clinical attention to moral aspects of psychotrauma. Despite abundant evidence clouded in personal experiences (1), persistent cognitions of shame and negative beliefs long remained a diagnostically unacknowledged phenomenon (2–4). Recent discourse around moral injury (MI) and moral distress (MD), however, has stimulated further consideration of these clinical observations (5). MI/MD refer to the psychosocial-spiritual harm associated with committing, failing to prevent, observing, or learning about an event that violates one's morals and values (6, 7). Such real or perceived transgressions or betrayals by self or others may cause harm to a person's wellbeing. MD/MI can have devastating impacts on the lives of many, leading to persistent guilt, social withdrawal and self-destructive behavior. While a better understanding of these constructs is needed, it is also important to advance the exploration of interventions that address the impacts of MI/MD on the human condition.

## Interdisciplinary collaborative treatment of moral injury and moral distress

There is much we do not know about both MI/MD and the domains within which they are situated. The field of psychiatry, for example, may be considering situating MI/MD within current diagnostic classifications; there may be support for a subtype of MI as part of PTSD (8). Other perspectives advocate for a broader interdisciplinary public health perspective of MI/MD (9, 10). There is a clear need to broaden the horizon to include domains such as morals and ethics, spirituality and religion, and philosophy and anthropology. An interdisciplinary approach is thought to be critical to bringing coherence to the discourse, laying the foundations for novel interventions and embedding diverse interventions into systems of care and support. Respectful interdisciplinary dialogue and exchange of ideas will be paramount to this endeavor. This Frontiers special topic: *Emerging treatments and approaches for moral injury and moral distress*, aims to address the imperative of finding evidence-based interventions that integrate interdisciplinary perspectives on MI/MD.

## Moral injury and moral distress: Contribution perspectives

The contributions comprising this e-collection of 14 papers cover a range of theoretical and practical important areas within the topic, including multi-partner perspectives from those with lived and living experience of MI/MD and those attempting to provide assessment, treatment and support.

In *Defining and assessing the syndrome of moral injury*, Litz et al. consider problems of Definition and assessment of MI with the Moral Injury Outcome Scale (MIOS) based on initial work of the MIOS consortium. The MIOS is a carefully constructed and promising instrument that makes an important contribution to the reliable and valid assessment of MI. The contribution of Easterbrook et al. in providing an analysis of *Risk factors for moral injury among Canadian armed forces personnel* begins to fill a significant gap in our knowledge regarding trauma-related factors associated with MI amongst military personnel in the Canadian context. Notably, the authors point to not only deployment-related factors but also child maltreatment as risk factors for MI.

In the face of COVID-19, we have become aware of the significance of MI/MD in relation to health care professionals. As such, it is appropriate that this e-collection contains contributions focusing on the healthcare environment. These contributions include an analysis of *Research gaps and recommendations to guide research on assessment, prevention, and treatment of moral injury among healthcare workers* authored by Maguen and Griffin, which stresses the importance of improved measurements, mixed methods approaches and conceptual clarity; and a scoping review of *Potential circumstances associated with moral injury and moral distress in healthcare workers and public safety personnel across the globe during COVID-19* by Xue et al., which focuses on providers' emotional response to moral dilemmas and challenges during the pandemic. A third contribution, by Smith-MacDonald et al., examines a promising e-health based intervention for this population: *Companions in the abyss: A feasibility and acceptability study of an online therapy group for healthcare providers working during the COVID-19 pandemic*.

The relevance of MI/MD to other diverse populations is also addressed. Extending to work concerning refugees, an ever-present group arising from natural disasters, war and/or political oppression, Mooren et al. discuss current evidence on *The impact of morally injurious events in a refugee population: a quantitative and qualitative study*. Two further papers examine and report MI/MD as it relates to serving police officers: *Moral injury in trauma-exposed, treatment-seeking police officers and military veterans: Latent class analysis* by Mensink et al.; and *Development of an online treatment module for support of treatment of moral injury in military veterans and police officers* by June ter Heide et al.. With respect to the latent class analysis paper, the authors report high PTSD severity in a comorbid MI-PTSD client group and indicate that there is a substantial subgroup of trauma-exposed, treatment-seeking police officers and military veterans that may suffer from MI. The June ter Heide et al. paper describes development of a favorably rated treatment module and outlines plans moving forward for further development and likely implementation into systems of care.

Other practical interventions are also included in this Research Topic. The contribution by Brémault-Phillips et al., in the data-reference-rich article outlining *Scenario-based supported interventions (SBSIs) for moral injury and PTSD: Data report of film and television references for use with uniformed professionals*, is a unique practical resource for facilitating dialogue on MI/MD prior to and following exposure to potentially morally injurious experiences. That practical offering fits with other intervention-focused contributions including *Companions in the abyss: A feasibility and acceptability study of an online therapy group for healthcare providers working during the COVID-19 pandemic* authored by Smith-MacDonald et al. and mentioned in the health care workers cluster, and two very important papers that, respectively, focus on Acceptance and Commitment Therapy and the use of deepfake technology in the context of “safe” perpetrator confrontation. *Case conceptualizing in acceptance and commitment therapy for moral injury (ACT-MI): An active and ongoing approach to understanding and intervening on moral injury* authored by Borges et al. outlines an approach to ACT-MI that may prove helpful as an intervention in this context. The *Initial development of perpetrator confrontation using deepfake technology in victims with sexual violence-related PTSD and moral injury* presented by van Minnen et al. is a fascinating approach using digital health technology that may also have other important applications in this context.

Following on from this excellent collection of original work, the reader has access to more theoretical considerations with the introduction of two novel models in *Toward a dual process model of moral injury and traumatic illness* by Barr et al. and *Caught between is and ought: The moral dissonance model* by Te Brake and Nauta. Within the former, the Dual Process Model is introduced with practical applications demonstrated through brief vignettes. This work postulates that approaches to treatment that entail principles of Stoicism, non-judgment of experience, acceptance, and values-oriented action, are more likely than traditional trauma treatment approaches to assuage MI. The latter work regarding the Moral Dissonance Model considers contextual factors associated with moral injury and proposes a framework akin to cognitive dissonance that may have explanatory power in this context. In *Forgiveness: A key component of healing from moral injury?* Brémault-Phillips et al. consider the impact of forgiveness on reconstituting moral identity, restoring relationships, and healing of body, mind and soul.

For the scholar accessing this area for the first time, or for more seasoned readers, this e-collection will further scholarly and interdisciplinary discourse on MI/MD. Our hope is that this e-collection will be a stimulus for increased engagement for the public good, help shape the field, and serve as a springboard for further critical conversations for appropriate interventions and treatments of MI/MD.

## Author contributions

All authors listed have made a substantial, direct, and intellectual contribution to the work and approved it for publication.
